# Recruitment of sublingual microcirculation using handheld incident dark field imaging as a routine measurement tool during the postoperative de-escalation phase—a pilot study in post ICU cardiac surgery patients

**DOI:** 10.1186/s13741-018-0091-x

**Published:** 2018-08-09

**Authors:** Zühre Uz, Can Ince, Philippe Guerci, Yasin Ince, Renata P. Araujo, Bulent Ergin, Matthias P. Hilty, Thomas M. van Gulik, Bas A. de Mol

**Affiliations:** 1 Department of Experimental Surgery and Translational Physiology, Academic Medical Center, University of Amsterdam, Meibergdreef 9, 1105 AZ Amsterdam, The Netherlands; 2 Department of Translational Physiology, Academic Medical Center, University of Amsterdam, Meibergdreef 9, 1105 AZ Amsterdam, The Netherlands; 3 Department of Cardio-Thoracic Surgery, Academic Medical Center, University of Amsterdam, Meibergdreef 9, 1105 AZ Amsterdam, The Netherlands

**Keywords:** Sublingual microcirculation, Incident dark field imaging, Cardiac surgery, Fluid overload, De-escalation, Fluid therapy

## Abstract

**Background:**

Management of tissue perfusion following cardiac surgery is a challenging task where common clinical parameters do not reflect microcirculatory dysfunction. Heterogeneity in blood flow perfusion and abnormalities in capillary density characterize microcirculatory dysfunction. The restoration of a normal microcirculation may become a novel target for therapy in the future in addition to macrocirculatory parameters. The aim of this study is to determine how the sublingual microcirculatory parameters vary at the bedside in post-cardiac surgery patients which underwent diuretic therapy to correct fluid overload.

**Methods:**

In this prospective observational pilot study, video clips of sublingual microcirculation in post-cardiac surgery patients receiving furosemide and/or spironolactone to achieve normal fluid balance were recorded using Cytocam-IDF imaging. Data was obtained on the first (T0), second (T1), and third (T2) day after the patients left the intensive care unit (ICU). Measurements were analyzed off-line to obtain the following microcirculatory parameters: total vessel density (TVD), microcirculatory flow index (MFI), proportion of perfused vessel (PPV), and perfused vessel density (PVD). Macrocirculatory parameters and body weight were also collected at these time points.

**Results:**

Ninety measurements were performed in ten post ICU cardiac surgery patients. Thirteen measurements were excluded due to quality reasons; these excluded measurements were spread across the patients and time points, and there was no loss of patients or time points. An increase in TVD was observed from T0 to T1 (20 ± 2.7 to 24 ± 3.2 mm/mm^2^; *p* = 0.0410) and from T0 to T2 (20 ± 2.7 to 26 ± 3.3 mm/mm^2^; *p* = 0.0005). An increase in PVD was present from T0 to T1 (19 ± 2.3 to 24 ± 3.5 mm/mm^2^; *p* = 0.0072) and from T0 to T2 (19 ± 2.3 to 26 ± 3.4 mm/mm^2^, *p* = 0.0008). Fluid overload was assessed through a positive cumulative fluid balance on the day of ICU discharge.

**Conclusions:**

Cytocam-IDF imaging to monitor microcirculation as a daily parameter is feasible and could become a valuable tool to non-invasively assess the tissue oxygenation at the bedside. An increase in TVD and PVD (functional capillary density) indicated the recruitment of the sublingual microcirculation in patients with diuretic therapy. Future research is needed to prove the correlation between the recruitment of the sublingual microcirculation and the de-escalation phase of the fluid management.

## Background

Cardiac surgery including or excluding cardiopulmonary bypass (CPB) is associated with several microcirculatory alterations and consequent reduction in tissue oxygenation (Kara et al. 2016). These alterations are due to many factors such as the activation of inflammatory and hemostatic systems, the surgery itself (Vranken et al. 2016), tissue trauma, anesthesia (Bernet et al. 2011; De Backer et al. 2009), hypothermia, hemodilution, and micro-emboli formation (Koning et al. 2014; Prestes et al. 2016).

Achieving and maintaining stable macrohemodynamics has always been crucial in critical care (McGuinness and Parke 2015). In spite of a rigid control of hemodynamic parameters, complications are frequently observed after cardiac surgery during the perioperative period in the intensive care unit and ward. A common perioperative complication is postoperative fluid overload.

Positive cumulative fluid balance due to excessive fluid administration during the perioperative period may cause complications such as respiratory distress, kidney failure, intra-abdominal hypertension (IAH), and impaired wound healing (Malbrain et al. 2014; Prowle et al. 2010; Xu et al. 2015) in post-intensive care unit (ICU) cardiac surgery patients (Haase-Fielitz et al. 2017) and in sepsis (Kelm et al. 2015; Loflin and Winters 2017; Vincent et al. 2006). As a result, morbidity is increased and quality of life after surgery (Ghaferi et al. 2009) is decreased.

At the microcirculatory level, the two consequences of excessive fluid administration jeopardizing microcirculation are (i) tissue edema with increased interstitial hydrostatic pressure, subsequent increase in the diffusion distance, and capillary collapse and (ii) hemodilution with decreased capillary hematocrit and functional capillary density (FCD) leading to decreased oxygen transport to the tissues (Ince 2015). This may lead to a loss of coherence between the macro- and microcirculation and tissue dysoxia (Arnemann et al. 2016).

Hemodilution results in lower hematocrit and blood viscosity, leading to a decrease in the oxygen-carrying capacity determined mainly by the number of red blood cells (RBCs). Despite the lower viscosity, this has been shown to ultimately decrease the FCD (Cabrales et al. 2006). Maintenance of adequate FCD is important for organ perfusion and function (Groner et al. 1999; Tsai et al. 1995). Several animal and human studies have reported that hemodilution decreases the FCD (Atasever et al. 2012; Ferrara et al. 2016; Turek et al. 2011). Atasever et al. demonstrated a decrease in the FCD in the sublingual microcirculation as an effect of the priming solution causing hemodilution during on-pump cardiac surgery (Atasever et al. 2011). Following this report, many clinical studies have shown that excessive fluid administration is associated with organ failure (Payen 2014; Vincent and De Backer 2013), which is why timely administration of de-escalation therapy is essential.

For these reasons, maintaining the fluid balance in the perioperative period is of utmost importance. The “de-escalation phase” is the event describing the removal of excess fluids from the patient (Frazee and Kashani 2016). Several options are available, but the common endpoint is fluid extraction by a step-wise increase in therapeutics from diuretics to renal replacement therapy (RRT) (Gandhi et al. 2012; Prowle et al. 2014; Xu et al. 2015). Administration of diuretics is the most common routine method for the de-escalation phase of the fluid overload in the cardiac surgical wards (Bellomo et al. 2001). One of the expected outcomes of the de-escalation phase is a decrease in tissue edema, recruiting microcirculation and leading to an increase in hematocrit associated with higher oxygen availability, which in turn recruits the microcirculation and increases the FCD by increasing blood viscosity. Both events promote oxygen transport to the tissues expected to support the metabolic needs of the organs. However, to date, no study has investigated the evolution of microcirculation during the de-escalation phase. With the advent of microcirculatory measurements at the bedside, monitoring the sublingual area may provide relevant information in terms of organ perfusion (Sheikh et al. 2009; Tanaka et al. 2015) and the consequences of excessive fluid on the microcirculation. To this end, we used incident dark field imaging (Cytocam-IDF; (Aykut et al. 2015; Hutchings et al. 2016; Massey et al. 2013)) to investigate the response of post-cardiac surgery microcirculation with respect to the de-escalation therapy. We hypothesized that (i) FCD can be assessed at the bedside, and (ii) FCD would increase during the de-escalation phase of fluid therapy.

In this pilot study, we introduced a potential new approach to postoperative patients by daily routine microcirculatory measurements during the de-escalation phase.

## Methods

### Patients

This prospective observational study was performed at the Academic Medical Center of the University of Amsterdam. Approval of the local Medical Ethical Review Committee (Reference number W15_076) was obtained. Patients were screened and informed during the preoperative period. The screening is applied by the ward doctor, and the patients are selected by the expectation to undergo diuretic (furosemide and/or spironolactone) therapy in the nursery ward. Non-consecutive patients who underwent cardiac surgery (ten patients with a 3-day follow-up: CABG *N* = 5, aortic valve surgery *N* = 4, combination CABG and aortic valve surgery *N* = 1) and were discharged from the ICU to the cardiac surgery ward with the expectation of undergoing a diuretic (furosemide and/or spironolactone) therapy, between September and October 2015 were included. The exclusion criteria were age < 18 years; pregnancy; inability to consent due to mental disorder; having maxillofacial trauma or ear, nose, throat tumors; and early discharge (hospital stay shorter than 3 days) from the nursery ward.

### Study design and measurements

After ICU discharge, sublingual microcirculatory measurements were performed in the cardiac surgery ward at three time points, within a few hours following ICU discharge (T0) and 24 h (T1) and 48 h (T2) after ICU discharge. For every time point, three image sequences at three different sublingual spots were recorded per patient by two trained professionals (ZU, YI). The mean of the three measurements is reported.

Sublingual microcirculatory measurements were performed using incident dark field (IDF) imaging (Cytocam™, Braedius Medical, Huizen, The Netherlands) (Gilbert-Kawai et al. 2016). Each clip was recorded at 25 frames/s for 4 s at the bedside and underwent a scan for exclusion quality criteria (Massey et al. 2013). The Cytocam-IDF is a third-generation handheld microscope that enables real-time in vivo visualization of the microcirculation. It consists of an illumination unit based on IDF imaging with a × 4 magnification lens. The illumination light is emitted with a short pulse time of 2 ms and a chosen wavelength of 548 nm, ensuring the highest absorption of oxyhemoglobin and deoxyhemoglobin, whereby red blood cells are visible within the vessels. The vessels that are not perfused will not be visible. The surrounding tissue mostly reflects light and is seen as a white area, allowing for a crystal clear identification of microcirculation (Gilbert-Kawai et al. 2016). The pulsing illumination light is synchronized with a computer-controlled image sensor, enabling image projection on a monitor. The new technology was recently validated in the study by Aykut et al. (Aykut et al. 2015), who compared Cytocam-IDF with Sidestream Dark-Field (SDF) imaging technology.

At time points T0, T1, and T2, hemodynamic parameters that are conventionally monitored in the cardiac surgery ward, such as heart rate, arterial blood pressure, and peripheral capillary oxygen saturation (SpO_2_), were acquired. Blood samples for hemoglobin, creatinine, hematocrit, and leukocyte count were collected according to the cardiac surgery ward care protocol at time points T0 and T1. Patient weight was measured the day before the operation and on the second and third days after ICU discharge in the post-cardiac surgery ward. Body weight was not measured on the first day in the ward, as the patients were still bedbound. Cumulative fluid balances at the end of surgery and the ICU stay were acquired.

### Measurements analysis

Following data collection, each video clip was assessed for adequate quality using the microcirculation image quality score proposed by Massey et al. (Massey et al. 2013). Automated Vascular Analysis (AVA) software v3.2 (MicroVision Medical, Amsterdam, The Netherlands) (Dobbe et al. 2008) was used to obtain microcirculatory parameters. Flow characteristics of the microvasculature were quantified using the microcirculatory flow index (MFI) according to the recommendations from the consensus on microcirculatory imaging by De Backer et al. (De Backer et al. 2007). This index was calculated after the image was divided into four quadrants, and the predominant type of flow (absent = 0, intermittent = 1, sluggish = 2, abnormal = 3, and hyperdynamic = 4) was estimated in the vessels smaller than 25 μm by the operator (Boerma et al. 2005). The final MFI score is a value obtained from the average score of the four areas. A quantitative measurement of the capillary density, the total vessel density (TVD; mm/mm^2^), was manually determined using AVA software. The proportion of perfused vessels (PPV; %) was automatically calculated by the software as the number of vessels with flow values of 2 and 3 divided by the total number of vessels. Perfused vessel density (PVD; mm/mm^2^) was determined as the total vessel density multiplied by the PPV. The PVD is used as a correct measure of FCD (De Backer et al. 2007). All video clips were analyzed off-line by the investigators (ZU, RP).

### Statistics

Statistical analysis was performed using GraphPad Prism 7.00 (GraphPad Software, La Jolla, CA, USA). A one-way analysis of variance was performed for microcirculatory parameters for a comparative analysis of the values obtained at each time point. Paired Student’s *t* test was used to compare the parameters collected at only two time points. All data are presented as the mean ± SD and considered statistically significant given a two-sided *p* < 0.05.

## Result

### Patient characteristics and systemic parameters

The main characteristics of the ten included patients are shown in Table 1. The patients received crystalloid (Sterofundin/NaCl 0.9%, B. Braun) fluid management, with the exception of one patient who received colloids in addition to the crystalloid. During the ICU stay, the patients received fluid management according to the guidelines of AMC on hemodynamic management, generally resulting in a cumulative positive fluid balance at the first ICU day. To account for this, the goal the following morning after surgery (postoperative day 1) was to reach a negative fluid balance. The period that the patients spent in the ICU ward was 1.6 ± 1.0 days, see Table 1. The goal of reaching a negative fluid balance was not reached during the patient’s stay in the ICU. The patients were discharged once they were hemodynamically stable and were sent to the ward to start the de-escalation phase to achieve a negative fluid balance. Patients received furosemide and spironolactone orally in the nursery ward.

**Table 1:** Patient characteristics

Patient characteristics	Mean ± SD
Age (years)	63.6 ± 9.9
Gender (male:female)	7:3
Preoperative weight (kg) (*N* = 9)	76.6 ± 12.5
BMI (kg/m^2^)	25.6 ± 3.9
Fluid balance at the end of the surgery (mL) (*n* = 7)	1425 ± 980
Cardiac surgery with CPB (%)	50
Duration of CPB (min)	105.2 ± 44.5
Duration of aortic clamping (min)	66.2 ± 26.3
Duration of the surgery (min)	146.6 ± 56.1
Period spent in the ICU (days)	1.6 ± 1.0
Period spent in the ward (days)	4.1 ± 2.1
Period spent in hospital in total (days)	6.6 ± 2.1
Cardiac surgery performed	
CABG (*N*)	5
AVR	
TAVI (*N*)	2
MIAVR (*N*)	1
MIAVR and valve papillary fibroelastoma resection combined (*N*)	1
CABG and AVR combined (*N*)	1

*BMI* body mass index, *CPB* cardiopulmonary bypass, *ICU* intensive care unit, *CABG* coronary artery bypass grafting, *AVR* aortic valve replacement, *TAVI* transcatheter aortic valve implantation, *MIAVR* minimally invasive aortic valve replacement

Systemic parameters were measured in all patients and are presented in Table 2. There was no significant change in the macrohemodynamic parameters between T0 and T2. The creatinine and hemoglobin levels and leukocyte counts are also presented in Table 2.

**Table 2:** Systemic parameters, diuretic medication, and blood results

Clinical systemic parameters	T0	T1	T2
Heart rate (bpm)	82.2 ± 7.6	81.2 ± 15.0	79.4 ± 14.2
Mean arterial pressure (mmHg)	88.1 ± 6.7	87.8 ± 13.7	89.2 ± 10.7
SpO_2_ (%)	93.9 ± 1.5	95.4 ± 2.4	95.8 ± 3.8
Temperature (°C)	37.2 ± 0.5	36.9 ± 0.5	36.7 ± 0.6
Furosemide (mg)	82.0 ± 147.4	67.0 ± 76.9	49.0 ± 73.1
Spironolactone (mg)	25.0 ± 16.7	22.5 ± 18.4	20.0 ± 19.7
Creatinine (μmol/L)	109.8 ± 121.3	102.9 ± 74.5	X
Hemoglobin (mmol/L)	6.9 ± 0.8	6.4 ± 0.8	X
Leukocyte (10^9^/L)	11.0 ± 3.9	10.4 ± 3.2	X

*X* not measured

One of the patients had postoperative acute kidney injury along with high plasma creatinine levels (T0, 450 μmol/L; T1, 307 μmol/L; T2, 167 μmol/L) and a consequently higher dose of furosemide (dose over 24 h) administered when compared to the rest of the sample group (T0, 500 mg; T1, 250 mg; T3, 250 mg). None of the patients received a blood transfusion or vasoactive medications during the post-ICU cardiac surgery nursery ward stay.

### Weight and cumulative fluid balance

**Fig. 1:**
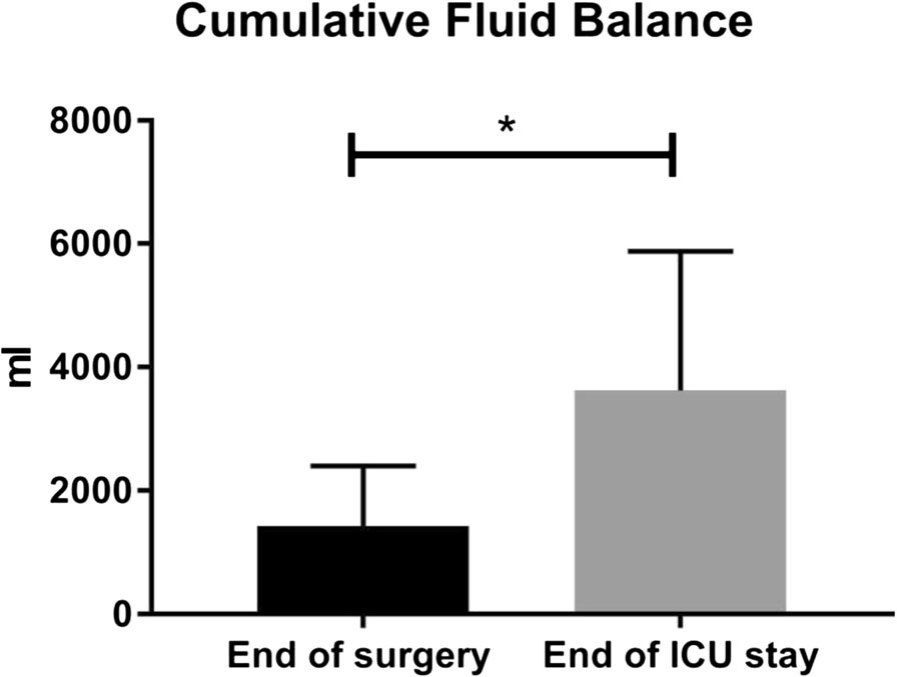
Cumulative fluid balance at the end of surgery and ICU stay. *p ≤ 0.05

The complete dataset of the weight was obtained in nine of the ten patients with the following values: 76 ± 12.6 kg in the preoperative phase, 78 ± 12 kg at T1, and 77 ± 11.7 kg at T2. No weight measurements were available at T0 because all patients were confined to bed after ICU discharge. There was a significant increase in the weight at T1 compared to the preoperative phase (*p* = 0.0065) and a significant decrease in the weight at T2 compared to T1 (*p* = 0.0193). In seven of the ten patients, the cumulative fluid balance was assessed, at the end of the surgery and the ICU stay. The cumulative fluid balance was 1425 ± 980 mL at the end of the surgery when patients arrived at the ICU ward. The cumulative fluid balance increased to 3625 ± 2251 mL at the moment of discharge from the ICU to the post-cardiac surgery nursery ward. This increase of the cumulative fluid balance during the ICU stay was significant (*p* = 0.0291), see Fig. 1.

### Feasibilty of Cytocam-IDF imaging

After the quality assessment of the video clips (Massey et al. 2013), 77 video clips were eligible for the analysis. The excluded clips were spread across the patients and time points, and there was no loss of patients or time points. In seven of the ten patients, there were 13 clips (13/90) excluded due to quality reasons. In five patients, there was only one clip excluded from each patient, and in two patients, there were four clips excluded from each patient. However, there was always at least one video clip at each time point to analyze the microcirculatory parameters. According to the quality assessment, only high-quality video clips are included which were blinded later for the analyses. The most important reason for the exclusion was the existing saliva in the sublingual cave that influenced the focus of the images, and thereby the sharpness of the individual RBCs. The saliva formed a layer between the organ surface and the tip of the device. Even though the microvessels could be identified with an altered focus, it was difficult to distinguish between the background vessels and the initially captured vessels. For these reasons, all the video clips with an altered focus due to excessive saliva are excluded.

### Microcirculatory parameters

**Fig. 2:**
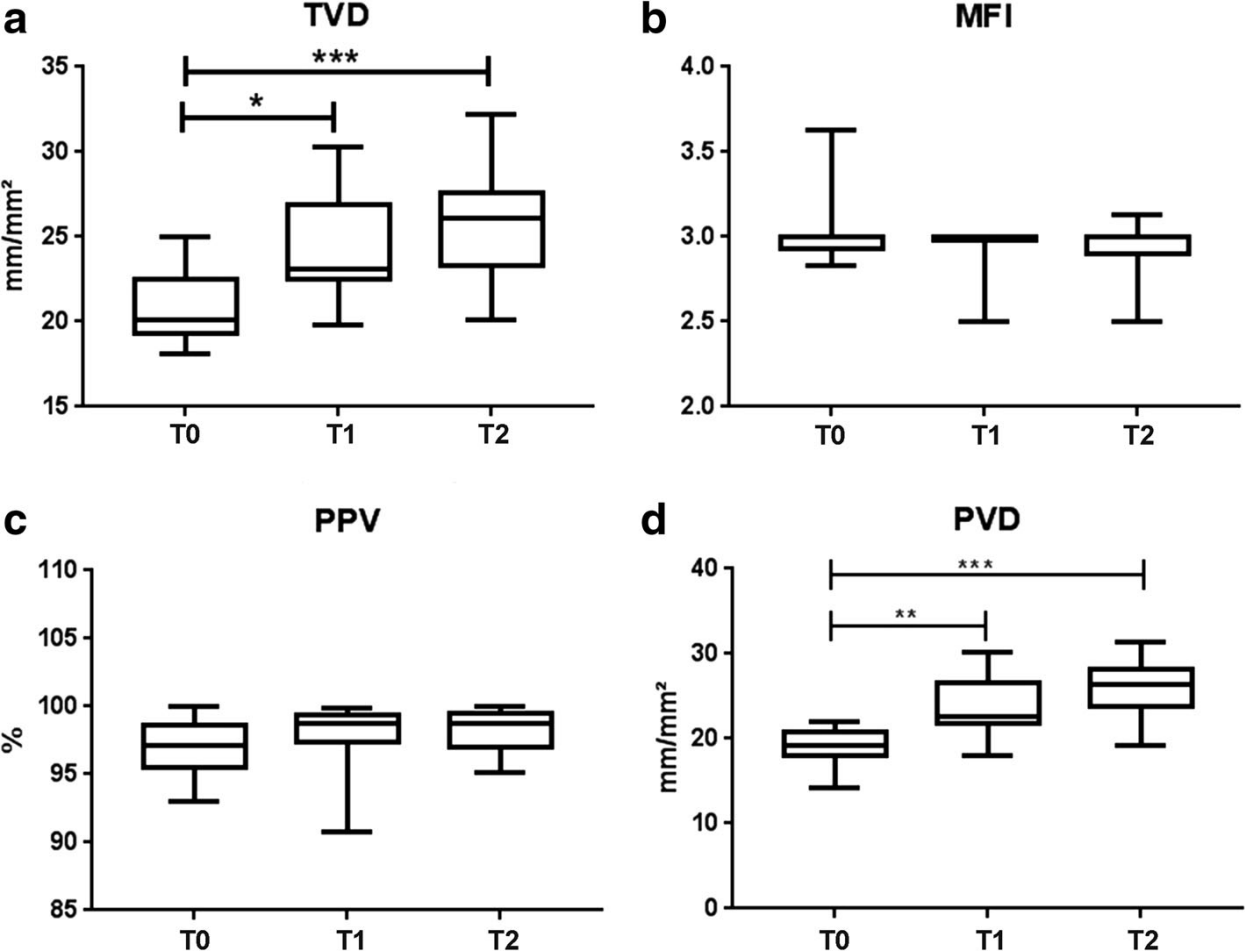
Microcirculatory parameters. Total vessel density (**a**), microvascular flow index (**b**), proportion of perfused vessels (**c**), and perfused vessel density (**d**) on the first (T0), second (T1), and third (T2) days after the ICU discharge. *p ≤ 0.05; **p ≤ 0.01; ***p ≤ 0.001

**Fig. 3:**
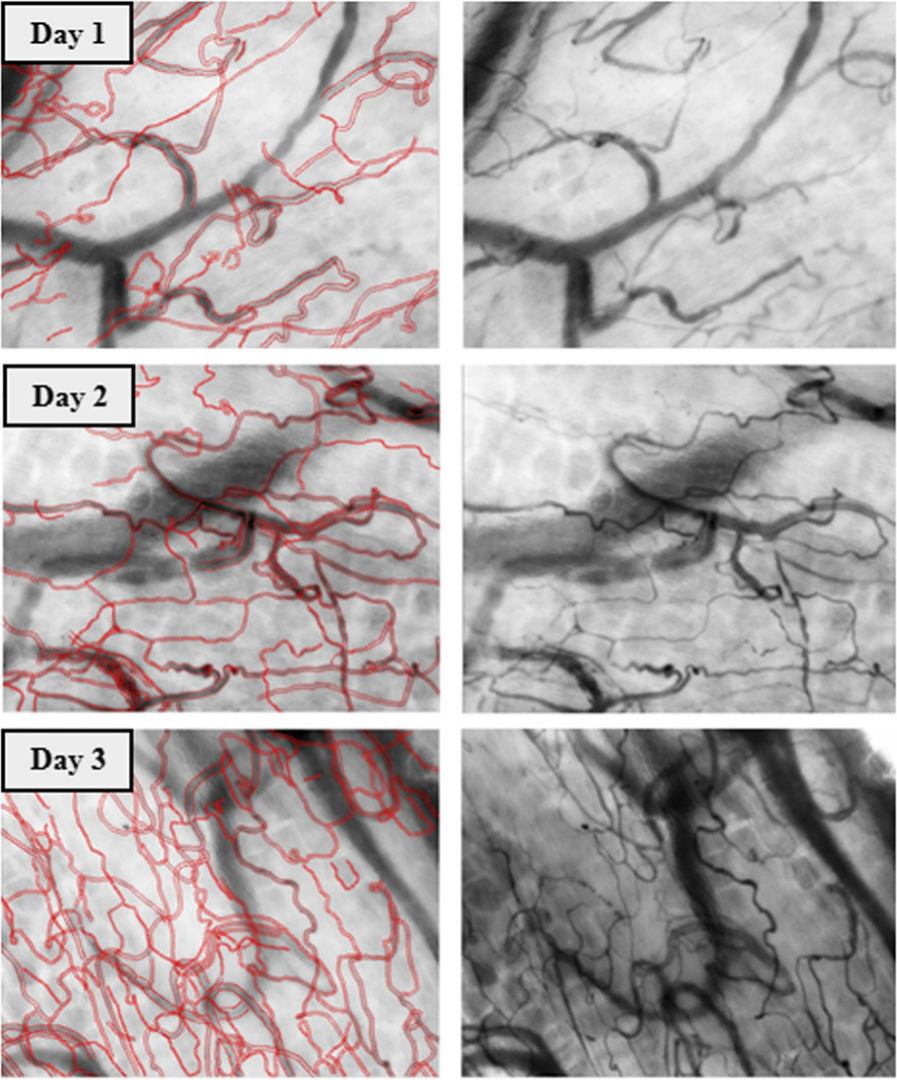
Cytocam-IDF images of sublingual microcirculation—a visual representation of the increase of the TVD. Images of sublingual microcirculation video clips obtained from one patient on day 1 (T0), day 2 (T1), and day 3 (T2) after ICU discharge. The red lines indicate the vessels smaller than 25 μm and represent the TVD within the entire image

Figure 2 shows the microcirculatory parameters for the 3-day follow-up. An increase in TVD was observed from T0 to T1 (20 ± 2.7 to 24 ± 3.2 mm/mm^2^; *p* = 0.0410) and T0 to T2 (20 ± 2.7 to 26 ± 3.3 mm/mm^2^; *p* = 0.0005). An increase in PVD was present from T0 to T1 (19 ± 2.3 to 24 ± 3.5 mm/mm^2^; *p* = 0.0072) and T0 to T2 (19 ± 2.3 to 26 ± 3.4 mm/mm^2^, *p* = 0.0008). There was no change in the microcirculatory flow parameters. A visual representation of the increase in the TVD over 3 days (T0, T1, and T2), from one random patient is shown in Fig. 3.

## Discussion

Our main findings were the (i) feasibility of a follow-up assessment of microcirculation using Cytocam-IDF in a post-cardiac surgery ward and (ii) the increase in the FCD during the follow-up in post-ICU cardiac surgery patients undergoing diuretic therapy. Although several studies have shown that fluid administration decreases FCD (Atasever et al. 2011, 2012; Turek et al. 2011; Yuruk et al. 2012), this is, to our knowledge, the first study demonstrating the conceivable beneficial effect of fluid de-escalation in recruiting microcirculation by increasing the FCD.

In the last decade, direct visualization of microcirculation has been performed mostly with orthogonal polarizing spectral (OPS) imaging and its successor, SDF imaging (Vellinga et al. 2010). Previous studies revealed that IDF imaging is feasible in different scenarios. Tanaka et al. proposed a real-time technique for the clinical assessment of microcirculation using IDF imaging, suggesting a possible clinical impact on the management of patients in the ICU (Tanaka et al. 2015). Van Elteren et al. demonstrated the feasibility of using Cytocam-IDF in the neonatal intensive care unit (van Elteren et al. 2015). Bedsides, SDF measurements were performed in cirrhotic patients with or without sepsis (Sheikh et al. 2009) in the intensive care unit as well as in a non-critical care context. Our study adds to these findings that a follow-up assessment of microcirculation during several days in the ward is feasible. We showed this feasibility by assessing 90 video clips during a 3-day follow-up with an exclusion of 13 video clips due to quality reasons. The most important reason for the exclusion was the excessive saliva in the sublingual cave. Nevertheless, this problem of excessive saliva could be solved by removing the saliva from the sublingual cave.

Currently, there is a trend to reduce the amount of fluids administered during the perioperative period in favor of a more tailored and titrated fluid administration. This has been emphasized in the past few years with the implementation of fluid-guided therapy to treat stroke volume variation during the surgery (Malbrain et al. 2014). Microcirculation might be jeopardized when the fluid is administered in excess. It would be beneficial during the administration of fluids (Ince 2014) to monitor microcirculation in order to optimize tissue perfusion. Although this idea has been implemented in several studies (Arnemann et al. 2016; Ince 2015; Malbrain et al. 2014), this has not until now been explored in the de-escalation phase (Malbrain et al. 2014).

Perioperative complications are currently monitored by the assessment of clinical and macrocirculatory parameters; these parameters are not finely tuned variables of ongoing changes in microcirculation, assuming that microcirculation itself can be the main factor in the pathogenesis of critical conditions. In the post-cardiac surgery ward, only blood pressure measurements by cuff are performed due to the unavailability of routine non-invasive cardiac output monitoring. Our study suggests that the recovery of FCD is at least partly associated with the removal of excess fluid and tissue edema. However, there are studies demonstrating that the recovery of the microcirculation does not depend on these factors alone (Koning et al. 2014; Malbrain et al. 2014; Romagnoli et al. 2016).

During surgery, especially CABG on-pump surgery, surgery-associated inflammation can occur and can also affect the microcirculation; this has been shown in an animal study, in which hemodilution altered the microcirculation, although hemodilution and inflammation had a cumulative effect (Koning et al. 2014; Romagnoli et al. 2016). Nevertheless, at T0, the patients were discharged from the ICU department after a stay duration of 1.6 ± 1.0 days, suggesting that a part of the inflammatory recovery of the microcirculation had already taken place. In addition, the fact that all the patients received furosemide therapy in combination with a positive cumulative fluid balance supports that the recruitment of the microcirculation was also partially caused by the de-escalation therapy.

Our study suffered some shortcomings. First, being a pilot study, the small sample size does not necessarily allow to generalize the finding. Seventy-seven video clips were analyzed, and this sample size was sufficient to demonstrate a significant difference in microcirculatory density (TVD) and perfusion (PVD). Second, the assessment of microcirculation recordings requires offline analysis, operator experience, and training, making bedside use impractical for now. Finally, no specific parameters, such as the daily net fluid balance or bioelectrical impedance analysis, were available in the cardiac surgery ward. These parameters would have provided additional information on the body fluid status of the patients. In the future study, these parameters will be measured to prove the correlation between the increase in FCD and the de-escalation phase of the fluid management.

## Conclusion

Cytocam-IDF imaging to monitor microcirculation as a daily parameter is feasible and could become a valuable tool to non-invasively assess the tissue oxygenation at the bedside. An increase in TVD and PVD (FCD) indicated the recruitment of the sublingual microcirculation. These findings suggest that IDF imaging may add to our understanding of the volume status in patients and could potentially guide clinicians in the management of the different fluid phases as proposed in several studies (Arnemann et al. 2016; Ince 2015; Malbrain et al. 2014).

## Abbreviations

AMC: Amsterdam Medical Center
AVA: Automated vascular analysis
AVR: Aortic valve replacement
BMI: Body mass index
CABG: Coronary artery bypass grafting
CPB: Cardiopulmonary bypass
CRRT: Continuous renal replacement therapy
FCD: Functional capillary density
IAH: Intra-abdominal hypertension
ICU: Intensive care unit
IDF: Incident dark field
MAP: Mean arterial pressure
MFI: Microvascular flow index
MIAVR: Minimally invasive aortic valve replacement
OPS: Orthogonal polarizing spectral
PPV: Proportion of perfused vessels
PVD: Perfused vessel density
RRT: Renal replacement therapy
SD: Standard deviation
SDF: Sidestream Dark-Field
SpO_2_: Oxygen saturation
TAVI: Transcatheter aortic valve
